# Patients’ thoughts on their falls in a rehabilitation hospital: a qualitative study of patients with stroke

**DOI:** 10.1186/s12877-021-02649-1

**Published:** 2021-12-18

**Authors:** Saika Aihara, Shin Kitamura, Masayuki Dogan, Sachiko Sakata, Kunitsugu Kondo, Yohei Otaka

**Affiliations:** 1 Department of Rehabilitation Medicine, Tokyo Bay Rehabilitation Hospital, Narashino, Chiba Japan; 2 Department of Rehabilitation, Faculty of Health Science, Tohoku Fukushi University, Sendai, Miyagi Japan; 3 Faculty of Rehabilitation, School of Health Sciences, Fujita Health University, Toyoake, Aichi Japan; 4 Department of Rehabilitation Medicine I, School of Medicine, Fujita Health University, 1-98 Dengakugakubo, Kutsukake, Toyoake, Aichi 470-1192 Japan

**Keywords:** Accidental falls, Cerebrovascular disorders, Patient participation, Patient safety, Rehabilitation

## Abstract

**Background:**

Patients with stroke in rehabilitation wards are at an increased risk of falling. Although patients’ participation in establishing medical safety is considered crucial, there is limited evidence on their perspectives of falls. This study aims to comprehensively elucidate the subjective falling experience of patients with stroke who have been admitted to rehabilitation wards.

**Methods:**

Twenty-three consecutive patients with stroke (44 to 90 years) who experienced a fall during hospitalisation were interviewed within 1 week after the fall, and thematic analysis was used to analyse the data.

**Results:**

Five themes surrounding fall events were extracted from the narratives: ‘Psychological background before the action’, ‘Support for the action’, ‘Direct causes of the fall’, ‘Patients’ awareness after the fall’, and ‘Changes in attitudes and behaviours after the fall’. ‘Psychological background before the action’ comprised hastiness or hesitation to call for help. Participants often took an action based on ‘Support for the action’ derived from their past experiences of moving safely, their confidence, and/or motivation to challenge themselves to move. ‘Direct causes of the fall’ consisted of unfamiliar actions, training fatigue, the surrounding environment, reduced physical function due to paralysis, lack of attention, overconfidence in their ability, and insufficient prediction of falls. ‘Patients’ awareness after the fall’ consisted of re-affirming difficult movements, the need for rehabilitation, a reduced ability to move, an increased risk of falling, the need for attention while moving, a fear of falling, and a lack of lessons learned from falling. Finally, patients demonstrated ‘Changes in attitudes and behaviours after the fall’ such as embodying a positive attitude to cope with the risk of falling or behavioural changes to reduce the risk of falling.

**Conclusions:**

Comprehensive information on patients’ perspectives before and after the fall was elucidated, uncovering many aspects including the psychological background for why patients engaged in risky behaviours resulting in falls, presence of positive thinking, and behaviour after the fall. By incorporating the patients’ views on fall incidences and their assessment, we can develop appropriate prevention strategies against falls.

## Introduction

Fall prevention in a hospital is an important issue in patient safety [1]. Falls are the most common incidents that occur in hospitals, accounting for approximately 30% of all incidents [2]. Falls can lead to physical complications such as fractures and trauma [3, 4], and are associated with high anxiety, depression, and low self-confidence [5], resulting in longer hospital stays and higher financial costs [6, 7]. The World Health Organization states that the increase in falls is a priority issue in aging countries and that ‘Fall prevention is a challenge to the ageing population’ in hospitals and facilities as well as in the community [8].

Patient participation is recognized as an important approach to ensure medical safety. The World Health Organization encourages patients and their families to actively participate in medical care to prevent adverse events and stay safe while in the hospital [9]. Educating patients by providing knowledge and sharing preventive strategies has been attempted to engage them in fall prevention practices at hospitals [10–13]. However, little attempt has been made to involve patients deeply in the process of fall assessment [14]. Looking back on patients’ thoughts and behaviours regarding their falls may help us understand the backgrounds, causes, and consequences of the fall.

Patients with stroke, in particular, are at a high risk of experiencing falls [15–19] because of multiple intrinsic risk factors, such as functional deterioration in motor, sensory, and cognitive abilities [16, 20, 21]. Higher fall rates have been especially reported among patients admitted to rehabilitation wards [18, 22–24]. The overall purpose of rehabilitation is to facilitate patients to independently engage in daily life activities again. However, increased physical activity can also increase the risk of falling [25]. Therefore, there is always a trade-off between independence and safety [26, 27]. Thus, there is a constant risk of falling at rehabilitation wards, and it is an important challenge to minimize this risk for patients recovering from a stroke while helping them regain their independence.

Few studies have investigated the experience of falls in hospitals from patients’ perspectives [28, 29]. Furthermore, to the best of our knowledge, no study has focused on the perspective of patients with stroke undergoing rehabilitation. Therefore, this study aimed to elucidate the subjective perspectives toward falling among patients with stroke admitted to rehabilitation wards.

## Methods

### Design

This qualitative study investigated patients’ thoughts before and after the fall, and their perception of the fall, by asking them to recall the fall in semi-structured interviews. A thematic analysis of the data was conducted. Thematic analysis involves analysing qualitative data and extracting common meanings, and then aggregating these to form a common theme [30, 31]. This is a suitable way to construct common themes from the semantic details included in the narratives of the participants. The protocol for this study was approved by the appropriate ethics committee and conformed to the Declaration of Helsinki.

### Study setting

The study was conducted in the Kaifukuki rehabilitation wards (KRWs) of a 160-bed convalescent rehabilitation hospital in Japan. The KRW is a system that provides subacute intensive rehabilitation covered by Japan’s medical insurance system, wherein patients with stroke are eligible for admission within 2 months of the onset of stroke symptoms and can stay up to 6 months after admission [32]. The individual risk of falling is assessed regularly among all patients, and necessary countermeasures are systematically implemented, such as using a wrist band for high-risk patients, supervision for/assistance in transfers/toileting/mobility, and appropriate environmental settings including mobility aids. Additionally, for patients at a higher risk of falls, specific safety measures (e.g., installation of sensors) are implemented based on the assessments and discussions performed by a multidisciplinary team.

### Participants

Participants were recruited between February 2016 and June 2017. Patients who had their first-ever stroke and experienced a fall while hospitalised in a KRW were consecutively recruited based on the hospital’s reports about their falls. As it was thought that they might not be able to properly describe the situation and their thoughts at the time of the fall in an interview, patients with aphasia and obvious cognitive deficiencies (MMSE < 24 points [33]) were excluded from the study. All patients provided written informed consent prior to their participation in the study.

### Data collection

With reference to a similar study [34], the interview guide questions (Table 1) were determined after multiple discussions among the authors, which included three occupational therapists (years of work experience: SA - 3, SK - 6, SS - 28) and one rehabilitation physician (YO: 20 years of work experience as a rehabilitation physician). The interview guide was used by one of the following three occupational therapist authors (SK, SA, MD – 2 years of experience) to conduct a semi-structured face-to-face interview with each participant, in a hospital private room with no disturbance, within a week of the fall. These occupational therapists did not have any relationship with the participants prior to the study. The interviewers followed an interview guide, and participants were asked to comment on their fall, focusing on the causes of falls, the awareness of falling risks before the fall, and how they viewed the fall incident. The interviews were audio-recorded, and an interviewer noted the behaviours of the participants during the interview, such as their facial expressions and gestures. In order to avoid large variations in the interview method, the occupational therapists who conducted the interviews agreed on the purpose of the interview and the method of using the interview guide prior to the interviews.

**Table 1:** Interview guide

**Category 1** [Patient’s thoughts on the cause(s) of the fall]	
•-Why do you think you fell? What do you think was/were the cause/causes of the fall? •-What were you trying to do when you fell? •-Can you tell me the details surrounding the situation when you fell?	
**Category 2** [Patient’s awareness of the risk of the movement when he/she fell]	
•-Were you always able to do the action that led to the fall? Did you think it was a safe movement? •-Did you move in a certain way for the first time? Did you try this movement during training or with assistance from someone else? •-Did you think this movement was risky or dangerous? Has anyone pointed out the risks or danger of this movement to you?	
**Category 3** [How the patient views the fall]	
•-Have you accepted the fall, or how do you view the fall? •-Did the staff or your family say something to you after you fell? •-What did you think/feel about what was told to you? •-Do you think the fall has affected you negatively somehow? If so, how? •-Do you think the fall has affected you positively somehow? If so, how? •-Has this fall changed the way you think about your own body? •-Has this fall changed the way you think about rehabilitation or hospital life?	

### Analysis

The audio-recorded interviews were transcribed verbatim by each interviewer; and a thematic analysis was conducted on a spreadsheet software using an inductive approach (Microsoft Excel for Mac 2016) [30, 31]. An author (SA) read the data entirely and coded the data such that one sentence was one unit, and then extracted codes that showed content related to the falls. During this process, notes on the participants’ behaviours during the interview were used to comprehend the meaning of the recorded narratives when it got difficult to interpret. After interviewing five participants, two authors (SA and SK) began to classify the codes according to similarity in the semantic content between the codes. The sub-themes were determined using the classified codes, and the themes were generated by aggregating them. To ensure that all codes were included in the generated sub-themes, the classification of codes was repeatedly modified, and the sub-themes and themes were modified. Following this, additional sampling and data collection were performed. Each time new data were acquired, the coded data were compared with the sub-themes and themes to check their suitability; the classification as well as the sub-themes and themes were also revised as necessary. In addition, to ensure the validity of the analysis, as part of researcher triangulation, a third author (YO), who was not involved in the code analysis, checked the sub-themes, themes, and codes to confirm their validity. If the third author (YO) thought that the analysis needed to be modified, the analysis was repeated, and the sub-themes and themes were modified until all three authors (SA, SK, and YO) reached an agreement through discussion. Data collection was stopped when three consecutive rounds of repeated data collection and analysis yielded no further themes or sub-themes. The authors SK and YO who worked on the analyses had prior experience with qualitative research.

## Results

Of the 480 patients with a first-ever stroke admitted to the hospital during the study period, 206 (245 events) experienced falls. Of these, we requested 26 patients who met the inclusion criteria to participate. Three patients declined to participate in the study because they did not want to speak about the fall(s) they experienced. Thus, the data of 23 patients were analysed. The participants’ mean age was 65.9 years (range: 44–90 years); 14 participants were male. The mean time from stroke onset to the fall was 92.3 days (range: 34–196 days), from admission to the rehabilitation hospital to the fall was 54.0 days (range: 0–146 days), and from fall to interview was 3.7 days (range: 1–7 days). The individual characteristics of the 23 participants are shown in Table 2.

**Table 2:** Overview of participants’ characteristics

Participant	Sex	Age, years	Disease	Duration between admission and falls, days	Duration after stroke onset, days	Duration after falls and interviews, days	Usual locomotion (Walking aid)	FIM Total score	Location of fall
1	Male	60	Infarction	0 (Same day)	41	2	Wheelchair	108	Around the bed
2	Female	83	Infarction	68	89	1	Walking (cane)	100	Around the bed
3	Male	74	Infarction	4	35	7	Walking	112	Around the bed
4	Female	63	Haemorrhage	11	61	3	Wheelchair	86	Corridor
5	Male	59	Haemorrhage	44	90	5	Wheelchair	107	Bathroom
6	Female	66	Haemorrhage	40	79	2	Wheelchair	107	Around the bed
7	Male	75	Haemorrhage	63	89	3	Walking (cane)	100	Around the bed
8	Male	85	Infarction	113	146	4	Walking	99	Around the bed
9	Male	76	Infarction	109	162	3	Walking (cane, orthosis)	79	Cafeteria
10	Male	44	Haemorrhage	110	157	3	Wheelchair	81	Around the bed
11	Male	72	Infarction	65	106	6	Walking (cane)	122	Around the bed
12	Male	55	Haemorrhage	3	34	3	Walking	124	Around the bed
13	Female	80	Infarction	39	69	6	Walking (cane)	115	Corridor
14	Female	81	Haemorrhage	49	90	2	Wheelchair	96	Around the bed
15	Female	83	Infarction	34	45	4	Wheelchair	71	Around the bed
16	Female	63	Haemorrhage	146	196	1	Wheelchair	86	Around the bed
17	Female	90	Infarction	89	117	4	Wheelchair	84	Bathroom
18	Male	42	Haemorrhage	2	46	6	Wheelchair	63	Around the bed
19	Male	50	Haemorrhage	112	137	4	Walking (cane)	96	Around the wash basin
20	Male	78	Haemorrhage	72	91	3	Wheelchair	93	Around the bed
21	Male	76	Infarction	26	50	3	Walking (cane)	122	Around the bed
22	Male	64	Haemorrhage	8	24	4	Walking	109	Bathroom
23	Male	58	Infarction	34	170	7	Walking (cane)	106	Around the bed

*FIM* Functional Independence Measure

The duration of the interviews ranged from 15 to 45 min (mean [SD] duration, 18 [9] minutes). None of the interviews were interrupted or discontinued. A total of 1231 codes were constructed from the data obtained. Of these, 269 codes were related to falls, and from these, we generated 22 sub-themes and five themes (Table 3).

**Table 3:** List of themes

Theme	Sub-theme	Examples of narratives	Data count
Psychological background before the action	Hastiness	We all feel hasty while going to the toilet because we want to relieve ourselves as quickly as possible. (P6)	10
	Hesitation to call for help	I’m reluctant (to call for the staff) because I know they are busy. What I dislike the most is to call for them and bother them. (P6)	9
Support for the action	Past experience of doing the action safely	I was fairly stable while doing a series of movements to sit on the toilet. (P5)	20
	Confidence to take the action	I thought it would be fine. (P12)	19
	Challenge to move	I tried to get it done myself. (P1)	4
Direct causes of the fall	Unfamiliar action	I am not used to riding in a wheelchair. (P4)	7
	Training fatigue	Rehab sessions have been getting harder lately, and I have increased accumulated fatigue in my left leg, which is the paralyzed side. (P10)	4
	Surrounding environment	I always felt that it is slippery here. (P4)	5
	Reduced physical function due to paralysis	I did not expect it to feel this heavy. (P6)	19
	Lack of attention	This is nobody’s fault but mine: I was a bit careless. (P9)	11
	Overconfidence in their own ability	I may have been a little overconfident while feeling that I have gained strength. (P2)	18
	Insufficient prediction of falls	I did not think I would fall. (P16)	9
Patients’ awareness after the fall	Re-affirming difficult movements	I found stepping back difficult. After the fall, I was convinced that I must not take a step back. (P2)	8
	Need for rehabilitation	If I get the time, I want to try rehabilitating this leg too, until completion. (P7)	4
	Reduced ability to move	I learned that I could easily get imbalanced, even due to (an easy) movement like this. (P6)	22
	Risk of falling	Similar things can happen when I go home. (P7)	8
	Need for attention while moving	I learned that I have to be attentive at all times. (P11)	18
	Development of fear of falling	After that (fall), even walking is tough. I’m scared when there are people and wheelchairs in my path. (P13)	1
	Lack of lessons learned from falling	It (falling) is not that big a deal, so I do not think much of it. (P18)	20
Changes in attitudes and behaviours after the fall	Positive attitude to cope with the risk of falling	I know I am bad at stepping back. I practised hard for these days (in rehabilitation training). It is also what Mr. X (the therapist) pointed out. (P2)	9
	Behavioural changes for reducing the risk of falling	I start (the movement) after imagining how I would carry out the movement. (P19)	35

### Overview of themes

**Fig. 1:**
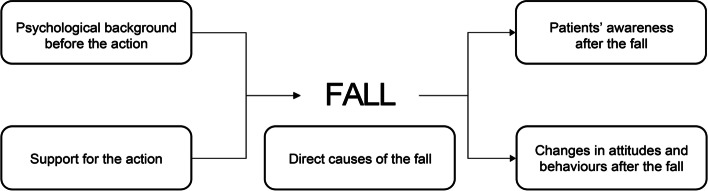
General conceptual diagram depicting the five themes

The following five themes were derived: ‘Psychological background before the action’, ‘Support for the action’, ‘Direct causes of the fall’, ‘Patients’ awareness after the fall’ and ‘Changes in attitude and behaviours after the fall’. The themes ‘Psychological background before the action’ and ‘Support for the action’ represented the patients’ psychological status and thoughts about taking the actions resulting in the fall. ‘Direct causes of the fall’ represented the participants’ opinions about the fall causes. ‘Patients’ awareness after the fall’ comprised things that participants became aware of after experiencing a fall, such as recognizing the risk of falling and the need for rehabilitation. ‘Changes in attitudes and behaviours after the fall’ centred around changes such as embodying a positive attitude to cope with the risk of falling, or behavioural changes to reduce the risk of falling (Fig. 1, Table 3).

### Psychological background before the action

This theme was mainly observed when a participant headed towards the toilet and hesitated to seek the aid of a staff member, whom the participant thought to be too busy to come and help him/her.

#### Hastiness

Participants carried out their intended actions in haste and with a sense of urgency. For example, ‘I was in a hurry then (to go to the toilet), so I was trying to get to the toilet as quickly as possible’ (P19).

#### Hesitation to call for help

Participants tended to hesitate to call for staff. ‘I am reluctant (to call for the staff) because I know they are busy’, (P6) and ‘What I dislike the most is to call for them and bother them’, (P6) showed that patients often hesitated to ask for help, because of their sympathy and concern for the staff.

### Support for the action

This theme was related to the participants’ thoughts on supporting themselves to take the action that led to the fall.

#### Past experience of doing the action safely

Past experiences of the participants, where they safely performed the action, which was eventually related to the fall, motivated them to do it again. One participant who had fallen while walking from near his bed to the bathroom, explained that the reason for taking the action that led to the fall was his past experience of doing the same without falling, during rehabilitation training and/or daily life with supervision. He said, ‘I had practised going to the toilet many times’ (P19).

#### Confidence to take the action

We documented how patients’ confidence to carry out an action also led to the fall. One participant had been moving around using a wheelchair because of difficulty in walking; however, she had attempted to walk by herself in her room and fell while trying to open the closet to take out some clothes. Regarding this action, she mentioned, ‘It was not a movement that I would worry about’, (the risk of falling) and ‘I thought I could do this movement because of that’ (not being worried) (P2). Another participant had always needed some assistance as she was at high risk of falling when being transferred to the toilet seat from her wheelchair. However, during the fall incident, she had gone to the bathroom and tried to transfer by herself. She stated, ‘Rather than being confident, I felt light-hearted, thinking I could probably do it’. (P14).

#### Challenge to move

Participants had experienced falls as a result of challenging themselves to perform risky movements. One participant fell twice while crouching. He practised the crouching movement with therapists after the first fall; but the second fall occurred when he attempted the same movement by himself in his hospital room. After the first fall, he stated ‘I thought about trying it’, (P8) indicating that he challenged himself to carry out the movement that he had learned through rehabilitation training with therapists.

### Direct causes of the fall

This theme was extracted from the narratives of the participants – from what they themselves felt were the direct causes for their falls.

#### Unfamiliar action

Participants had little experience with the movements and explained that a lack of familiarity was the cause of their falls. One participant explained that she fell off the wheelchair while extending her hands down toward her feet, as she was ‘not used to riding (in) a wheelchair’, (P4); this referred to how the fall was caused by her lack of familiarity with a motion different from what she was accustomed to, before the onset of stroke. Another participant had fallen while walking from the vicinity of the wash basin to the bathroom; he mentioned, ‘I didn’t have much experience going to the bathroom from the wash basin’, (P19) indicating that he did not have much practice of the movement.

#### Training fatigue

Some participants believed that fatigue from rehabilitation training just before the fall and/or the accumulation of fatigue from everyday training was the cause for the fall. One participant who fell while walking, recalled that there was pain in his leg at the time of the fall. He said, ‘I over-trained my right leg’ (the paralyzed side) (P8). Another participant recalled, ‘Rehab sessions have been getting harder lately, and I have increased the accumulated fatigue in my left leg, which is the paralyzed side’ (P10).

#### Surrounding environment

Participants also mentioned that their environment was the cause for the fall. One participant fell while transferring himself from the toilet seat to the wheelchair in the bathroom; he recounted, ‘I always felt that it is slippery here (the bathroom floor)’, (P5) thereby citing the slipperiness of the bathroom floor as the cause for his fall. Another participant who fell during the night explained, ‘I was not able to put on shoes during the night and therefore only wore socks; this made it difficult for me to plant my feet on the ground’ (P10).

#### Reduced physical function due to paralysis

According to the accounts of the participants, reduced physical function due to hemiparesis, such as the inability to move their limbs the way participants want, and heaviness of the arms had caused their falls. One participant fell while sitting on the wheelchair and leaning forward to try and pick up her spectacles’ case that had fallen on the floor. She explained, ‘I thought this half (the paralyzed side) would move a little more, I did not expect it to feel this heavy’, indicating the heaviness of the paralyzed arm as the cause for the fall (P6).

#### Lack of attention

Participants’ lack of attention while engaging in a movement and their inattention toward their own bodies were cited as probable causes for their fall. One participant mentioned that she failed to recognize her own body. This is evidenced by her comment, ‘I did not recognize my leg (on the affected side)’, indicating that to be the probable cause of the fall (P4). Another participant had fallen while trying to sit on the bed, and he commented, ‘I should have checked (the sitting posture) carefully’ (P21).

#### Overconfidence in their own ability

Participants mentioned their confidence in carrying out a movement. However, on looking back on their actions at the time of the fall, they recognized that they were overconfident. One participant looked back on his fall in the bathroom and recalled, ‘I was probably overconfident’ (P5). Another participant also mentioned the element of overconfidence in her comment, ‘I may have been a little overconfident while feeling (that) I have gained strength’, (P2) suggesting that overconfidence resulting from rehabilitation and practical abilities gained in daily life caused the fall.

#### Insufficient prediction of falls

Participants described that they had failed to adequately predict the outcome of moving with paralyzed limbs and fell as a result of not being able to cope with the unexpected outcomes of the movement. One participant mentioned, ‘I did not imagine I would fall’, (P4) while another participant recalled, ‘No, I was moving without thinking about falling’ (P7). Further, another participant recounted, ‘I have never been paralyzed before, and I did not have an understanding of what would happen if I move(d)’ (P6).

### Patients’ awareness after the fall

This theme comprised what the participants became aware of as a result of experiencing a fall.

#### Re-affirming difficult movements

Participants recognized movements that were difficult for them through their experience of falling. One participant recounted her fall by stating, ‘I found stepping back difficult’ and ‘After the fall, I was convinced that I must not take a step back’ (P2).

#### Need for rehabilitation

Participants recognized the importance of engaging in rehabilitation. One participant fell while transferring himself from the wheelchair. He spoke about the decline in his physical strength while recounting his fall and said, ‘I think I need rehabilitation’ (P1). Another participant had fallen while taking a step back while moving things in and out of the closet. She mentioned her rehabilitation training where similar movements were introduced by her therapist after the fall, ‘This (training of difficult movements) is beneficial for me. It was very helpful’ (P2). She seemingly recognized the importance of ensuring the safety of movements and continuing the rehabilitation.

#### Reduced ability to move

Participants recognized through their experience of the falls that their physical functionality had deteriorated from their premorbid status. One participant recounted his experience of falling while trying to put on his shoes by stating, ‘(What I noticed is) How much my physical strength deteriorated’ (P1). Another participant stated, ‘I learned that I could easily get imbalanced, even due to a movement like this’, (P6) indicating that the experience of falling helped her understand that certain movements would lead her to lose her balance.

#### Risk of falling

Participants recognized the risk of falling through their experiences of the falls. One participant fell while walking around in his hospital room. He mentioned the risk of falling at home after being discharged from the hospital, ‘Similar things can happen when I go home’ (P7). Another participant too recognized the possibility of falling again upon reflecting on his physical capacity by stating, ‘I tend to fall easily because of my impaired body condition’ (P12).

#### Need for attention while moving

After falling, participants recognized the need for paying attention while moving from one place to another. One participant recounted his experience of falling when he tried to stand up while sitting on the bed by stating, ‘What I learned is that I have to be a little more careful (with my movements) and ensure safety’ (P20). Another participant commented, ‘It is necessary to always be attentive because even if I think it is safe, I never know what is going to happen’ (P21) thereby recognizing the possibility of unexpected circumstances even when one is confident about performing certain movements safely, and the need to always be attentive.

#### Development of fear of falling

Participants had developed a fear of falling after their experience of the fall. One participant commented, ‘After that (fall), even walking is tough. I’m scared when there are people and wheelchairs in my path’ (P13).

#### Lack of lessons learned from falling

While the medical staff recognized a fall as a serious event, some participants thought it was not a matter of grave importance. One participant fell while trying to transfer from the wheelchair to the bed by himself when the nurse had taken her eyes off him for a second. His comment was, ‘It (falling) is not that big a deal’, (P18) indicating that he does not consider falling to be a serious or threatening incident. Another participant recounted his experience saying, ‘I just landed on my bottom, so I don’t consider it a fall’, (P11) suggesting that he did not consider an incident as a fall unless he sustained injuries.

### Changes in attitudes and behaviours after the fall

This theme centred around changes in the participants’ thoughts, attitudes, and behaviours after the fall.

#### Positive attitude to cope with the risk of falling

Some participants expressed a positive attitude to cope with the risk of falling after the fall incident. One participant had fallen backward while stepping back to open and close the closet door. She commented, ‘I know I am bad at stepping back. I practised hard for these days (in rehabilitation training). It is also what Mr. X (the therapist) pointed out’, (P2) indicating that she was sharing with the therapist, the movements which involved the risk of falling and training hard at them during the rehabilitation sessions. Another participant, on the other hand, commented on a poster calling for attention about dangerous and risky movements, ‘That would help prevent falls’ (P20). He commented positively mentioning that posters would be a good way to alert patients to be aware of the risk of falls in the future.

#### Behavioural changes for reducing the risk of falling

Participants had modified their behaviours after the fall to avoid falling again. One participant commented ‘I start (the movement) after imagining how I would carry out the movement’ (P19). The experience of falling led him to take steps to avoid sudden movements and envision the action prior to its execution. Another participant, on the other hand, was required to use a cane while walking around in his hospital room, but he mentioned, ‘It was cumbersome to use it every time I went to the toilet, so at times I walked small distances without the cane’. Before the fall, he was walking around without using his cane. However, the fall brought about changes in his behaviour: ‘I started to walk using a cane, whether it is to go to the toilet or to go brush my teeth. This was the lesson I learned’ (P9).

## Discussion

This study successfully elucidated the subjective experience of falls of patients with stroke admitted to a rehabilitation hospital into five main themes.

Few existing studies have examined patients’ subjective perceptions of falls in hospitals. A study conducted on patients with various diseases in a tertiary referral hospital [28] identified the following themes: 1) patients’ feelings of safety during hospitalisation, 2) realizing the risk of falling after the fall, 3) regaining their independence and identity including consideration of strategic ways to mitigate the risk of falling. Similarly, a small sample study (*n* = 5) with patients in rehabilitation wards [29] derived the following themes: 1) patients’ perceptions about the causes of their fall, 2) their thoughts about changes in the way they move around the hospital after the fall, 3) decreased confidence and negative attitudes toward rehabilitation, and 4) changes in the role of staff. In a study conducted in an acute care hospital with inpatients of various diseases, two themes were identified: 1) reasons for falling and 2) patient activities to reduce falls. Under reasons for falling, patients reported losing their balance owing to an urgent need to reach the bathroom and unexpected weakness; under patient activities to reduce falls, patients reported positive attitudes such as changing their behaviour and seeking communication with medical staff to prevent recurrence of falls [35].

In the current study, we presented a more comprehensive view of patients’ perspectives of their falls than previous studies [28, 29, 35]. Particularly, this study conceptualized, for the first time, a chronological series of patients’ thoughts on their falls, starting from the patients’ psychological state before the fall to their behavioural change after the fall. In addition, a robust study design, considering data saturation and confirming the validity of the analysis through triangulation, resulted in a greater variety of valid themes being extracted. Furthermore, this study was conducted among patients with stroke, who underwent rehabilitation, thereby, uncovering several aspects regarding the subjective perception of falls among these patients during their recovery. The contents of each theme emerging in this study have been discussed as follows:

### Psychological background before the action

This theme described the psychological background—potential factors leading to the risky behaviour resulting in the fall—of patients prior to the fall. The two types of psychological backgrounds described in this theme have been reported in previous studies among patients with a wide variety of diseases [35, 36] and are considered to be common pre-fall psychological states experienced by hospitalized patients, which are not specific to patients with stroke. For example, when participants found themselves in an urgent situation such as the need to urinate, they were required to make a judgment call. In such situations, participants acted impatiently, anxiously, and poorly judged their physical ability, thereby prompting instinctual action, where they perceived their bodies to be in the condition prior to the onset of the stroke. Additionally, they were reluctant to ask for help, out of consideration for the busy staff; thus, they acted on their own when the need to urinate or defecate arose, despite requiring assistance in their state.

### Support for the action

Emotions such as the sub-themes of ‘Past experience of doing the action safely’ and ‘Confidence to take the action’ resulting from the participants’ past experiences were considered to be the direct motivations for the actions that resulted in the falls. Regarding ‘Challenge to move’, a qualitative study examining experiences of individuals with stroke, who had fallen, reported their desire to be independent, which caused them to perform movements despite their awareness of the risk of falling [37]. Other qualitative studies examining the recovery experience post a stroke indicated a typical behaviour known as ‘Experiencing the possibilities’, wherein individuals with stroke took actions involving risks while evaluating their ability to move amidst performing activities of daily living [38, 39]. A previous study examining fall-related experiences of individuals with stroke revealed that their confidence increased by pursuing the challenge of moving [40]. Moreover, in a study of older patients admitted to tertiary or rehabilitation hospitals, ‘Testing physical boundaries’ was classified as the reason for risk-taking behaviour that may lead to a fall [41]. Thus, challenging tasks have positive aspects. Its importance lies in devising strategies to minimize the risk of falling while implementing this positive psychological aspect in rehabilitation.

### Direct causes of the fall

This theme comprises seven sub-themes, which participants considered as the direct causes of their falls. While many studies have investigated the circumstances, risk factors, and consequences of falls [42], only few have investigated hospitalized patients’ thoughts on direct causes of their falls. These reports identified loss of balance [29, 35], unexpected weakness [35], and overoptimistic self-assessment [36] as subjective thoughts on the direct causes of falls. Another study of community-dwelling individuals with chronic stroke reported several perceived causes including balance deficits, muscle weakness, fatigue, confidence, and surrounding environments [43]. Majority of the causes revealed in our study are consistent with previous studies [29, 35, 36, 43], except for two new aspects—‘Unfamiliar action’ and ‘Insufficient prediction of falls’. This may have manifested because of the different characteristics of the sample in our study compared to the previous studies. In the current study, the majority of participants experienced a stroke within past 6 months and were in subacute phase. Furthermore, all participants were undergoing rehabilitation and their symptoms and abilities were in the process of recovery. This may have made it more difficult for them to adapt to their physical situation compared to patients with chronic stroke who were in a stable condition for a long time. Therefore, the participants in this study may not have fully understood their physical limitations and the risks of falling. Examining patients’ perceived experiences of falling and asking them to share their thoughts regarding the causes of their falls may lead to the development of more appropriate interventions.

### Patients’ awareness after the fall

Sub-themes such as ‘Re-affirming difficult movements’, ‘Reduced ability to move’, and ‘Need for attention while moving’ suggest that participants regained awareness of their bodies and surrounding environment through their experiences of falling; they also realized the need to pay more attention than before. In addition, participants’ realization regarding the risks of falling and the need for rehabilitation indicates their efforts to understand their bodily changes after the stroke. Thus, the participants of this study became aware of several things after the fall, which included multiple positive aspects. Previous studies have also shown that patients with stroke tend to gain a deeper understanding of their condition and abilities through the experience of falling [37, 40, 44], and become more careful when performing movements [37, 43]. Contrarily, participants also declared their fear of falling again in the future. It has been reported that fear of falling among individuals with stroke limits their mobility; thereby, further reducing physical function, and leading to decreased physical activity [44, 45]. This study’s findings support this result that post-fall fear is one of the negative consequences of falling. As described above, the present study shows that falls can influence patients positively as well as negatively.

### Changes in attitudes and behaviours after the fall

This theme described the post-fall changes in the patients’ state of mind, as well as the preventive strategies employed in movement and training therapy to avoid falling again. Participants exercised careful attitudes and modified their behaviours based on their observations about their own condition after the fall. Switching to practising safer movements and increasing training to overcome difficult movements are considered important in preventing recurring falls. This theme is supported by existing findings establishing that patients begin to implement new movement strategies tailored to their body, after the stroke, based on their experience of falls and attempts at risk-taking behaviours [34, 40, 43, 46].

### Limitations

This study had certain limitations. As the data were obtained by conducting interviews, only the data from participants who maintained relatively good cognitive function could be collected; thus, the study did not consider the opinion of patients with stroke as a whole. In addition, all the data in the study were collected from a single facility, and all the participants were Japanese. Therefore, generalization should be considered with caution when applying the findings to patients in different situations and countries. Furthermore, the results of the present study may reflect the characteristics of patients with stroke, such as perceptual changes after stroke. However, there was no age-matched comparison group of older people to determine whether the findings were specific to patients with stroke. A future comparison study with age-matched older people will explore the stroke-specific themes, and enable the suggestion of more disease-specific approaches, such as interventions against fall risk associated with altered perception in stroke. Despite the above limitations, we believe that this study provides important knowledge to help understand patients’ perspectives regarding their fall experiences.

## Conclusions and clinical implications

The themes extracted in the present study will provide valuable information to help establish better strategies to prevent falls among patients with stroke in rehabilitation wards. In particular, by using a temporal framework, this study was able to provide suggestions on when and how to interact with patients to prevent falls at the hospital and individual level.

Regarding pre-fall interventions, it is important to provide an appropriate environment that makes it easy for a patient to call the medical staff for help, and also create an environment for patients to act safely and avoid a situation in which they feel hasty or impatient. Furthermore, the present study tells us, at least partially, why there is a large number of falls resulting from patients attempting movements that are beyond their capacity [23]. Past qualitative research has shown that individuals with stroke need support that respects their sense of identity and their desire for independence [37]. Therefore, considering a patient’s desire to challenge new movements, and providing opportunities to attempt these movements under staff supervision or assistance can help patients confirm their limits of abilities, and may help reduce the risk of falling by attempting risky actions.

Regarding post-fall interventions, decisions are often made only by medical staff when taking fall prevention measures for patients or analysing falls that have occurred [14]. However, there is a limit to how much medical staff can know about the psychological background of a fall, as described by the participants in this study. To better match interventions for falls with patient behaviour, at the individual level, it is necessary to ask patients about the circumstances of their fall and then share with them strategies on how to prevent recurrence of the fall. At the hospital level, it is necessary to establish a system that involves patients, for example, by including their perspectives in incident reports.

In conclusion, the study elucidated five important themes expressing patients’ subjective experience, uncovering many aspects including the presence of positive thinking and behaviours after the fall, and the psychological background for why patients took risky behaviours resulting in the fall. By incorporating patients’ views on fall incidence and its assessment, we can develop appropriate prevention strategies against falls.

## Data Availability

The datasets used and/or analysed during the current study are available from the corresponding author on reasonable request.

## Abbreviations

KRW: Kaifukuki Rehabilitation Wards
MMSE: Mini-Mental State Examination

## References

[1] Joint Commission International. https://www.jointcommissioninternational.org/. Accessed 27 Oct 2020.

[2] Healey F, Scobie S, Oliver D, Pryce A, Thomson R, Glampson B (2008). Falls in English and welsh hospitals: a national observational study based on retrospective analysis of 12 months of patient safety incident reports. Qual Saf Heal Care.

[3] Tinetti ME, Speechley M, Ginter SF (1988). Risk factors for falls among elderly persons living in the community. N Engl J Med.

[4] Campbell AJ, Borrie MJ, Spears GF, Jackson SL, Brown JS, Fitzgerald JL (1990). Circumstances and consequences of falls experienced by a community population 70 years and over during a prospective study. Age Ageing.

[5] Scheffer AC, Schuurmans MJ, van Dijk N, van der Hooft T, De Rooij SE (2008). Fear of falling: measurement strategy, prevalence, risk factors and consequences among older persons. Age Ageing.

[6] Wong CA, Recktenwald AJ, Jones ML, Waterman BM, Bollini ML, Dunagan WC (2011). The cost of serious fall-related injuries at three midwestern hospitals. Jt Comm J Qual Patient Saf.

[7] Morello RT, Barker AL, Watts JJ, Haines T, Zavarsek SS, Hill KD (2015). The extra resource burden of in-hospital falls: a cost of falls study. Med J Aust.

[8] World Health Organization (2007). WHO Global Report on Falls Prevention in Older Age.

[9] World Health Organization (2013). Exploring patient participation in reducing health-care-related safety risks.

[10] Haines TP, Bennell KL, Osborne RH, Hill KD (2004). Effectiveness of targeted falls prevention programme in subacute hospital setting: randomised controlled trial. Br Med J.

[11] Haines TP, Hill AM, Hill KD, McPhail S, Oliver D, Brauer S (2011). Patient education to prevent falls among older hospital inpatients: a randomized controlled trial. Arch Intern Med.

[12] Hill AM, McPhail SM, Waldron N, Etherton-Beer C, Ingram K, Flicker L (2015). Fall rates in hospital rehabilitation units after individualised patient and staff education programmes: a pragmatic, stepped-wedge, cluster-randomised controlled trial. Lancet.

[13] Ang E, Mordiffi SZ, Wong HB (2011). Evaluating the use of a targeted multiple intervention strategy in reducing patient falls in an acute care hospital: a randomized controlled trial. J Adv Nurs.

[14] de Jong LD, Francis-Coad J, Waldron N, Ingram K, McPhail SM, Etherton-Beer C, et al. Does free-text information in falls incident reports assist to explain how and why the falls occurred in a hospital setting? J Patient Saf. 2021;17:e1472–9. 10.1097/PTS.0000000000000533 30192260

[15] Nyberg L, Gustafson Y (1995). Patient falls in stroke rehabilitation a challenge to rehabilitation strategies. Stroke.

[16] Tutuarima JA, van der Meulen JHP, de Haan RJ, van Straten A, Limburg M (1997). Risk factors for falls of hospitalized stroke patients. Stroke.

[17] Jorgensen L, Engstad T, Jacobsen BK (2002). Higher incidence of falls in long-term stroke survivors than in population controls: depressive symptoms predict falls after stroke. Stroke.

[18] Lee JE, Stokic DS (2008). Risk factors for falls during inpatient rehabilitation. Am J Phys Med Rehabil.

[19] Rapp K, Ravindren J, Becker C, Lindemann U, Jaensch A, Klenk J (2016). Fall risk as a function of time after admission to sub-acute geriatric hospital units. BMC Geriatr.

[20] Nyberg L, Gustafson Y (1997). Fall prediction index for patients in stroke rehabilitation. Stroke.

[21] Sze KH, Wong E, Leung HY, Woo J (2001). Falls among Chinese stroke patients during rehabilitation. Arch Phys Med Rehabil.

[22] Suzuki T, Sonoda S, Misawa K, Saitoh E, Shimizu Y, Kotake T (2005). Incidence and consequence of falls in inpatient rehabilitation of stroke patients. Exp Aging Res.

[23] Hanger HC, Wills KL, Wilkinson T (2014). Classification of falls in stroke rehabilitation - not all falls are the same. Clin Rehabil.

[24] Czernuszenko A, Czlonkowska A (2009). Risk factors for falls in stroke patients during inpatient rehabilitation. Clin Rehabil.

[25] Lord SR, March LM, Cameron ID, Cumming RG, Schwarz J, Zochling J (2003). Differing risk factors for falls in nursing home and intermediate-care residents who can and cannot stand unaided. J Am Geriatr Soc.

[26] Tinetti ME, Kumar C (2010). The patient who falls: “It’s always a trade-off.”. JAMA.

[27] Rubenstein LZ, Josephson KR, Trueblood PR, Loy S, Harker JO, Pietruszka FM (2000). Effects of a group exercise program on strength, mobility, and falls among fall-prone elderly men. J Gerontol Med Sci Public Domain.

[28] Gettens S, Fulbrook P, Jessup M, Choy NL (2018). The patients’ perspective of sustaining a fall in hospital: a qualitative study. J Clin Nurs.

[29] Turner N, Jones D, Dawson P, Tait B (2019). The perceptions and rehabilitation experience of older people after falling in the hospital. Rehabil Nurs.

[30] Braun V, Clarke V (2006). Using thematic analysis in psychology. Qual Res Psychol.

[31] Clarke V, Braun V (2013). Teaching thematic analysis: overcoming challenges and developing strategies for effective learning. Psychologist.

[32] Miyai I, Sonoda S, Nagai S, Takayama Y, Inoue Y, Kakehi A (2011). Results of new policies for inpatient rehabilitation coverage in Japan. Neurorehabil Neural Repair.

[33] Folstein MF, Folstein SE, McHugh PR (1975). “Mini-mental state”. A practical method for grading the cognitive state of patients for the clinician. J Psychiatr Res.

[34] Makino M, Izumi K, Hiramatsu T (2010). Thinking processes for patients with hemiplegia experiencing the possibilities of events leading to fall. J Tshruma Heal Sci Soc Kanazawa Univ.

[35] Carroll DL, Dykes PC, Hurley AC (2010). Patients’ perspectives of falling while in an acute care hospital and suggestions for prevention. Appl Nurs Res.

[36] Hill AM, Francis-Coad J, Haines TP, Waldron N, Etherton-Beer C, Flicker L (2016). “My independent streak may get in the way”: how older adults respond to falls prevention education in hospital. BMJ Open.

[37] Walsh ME, Galvin R, Williams DJP, Harbison JA, Murphy S, Collins R (2019). The experience of recurrent fallers in the first year after stroke. Disabil Rehabil.

[38] Doolittle N (1992). The experience of recovery following lacunar stroke. Rehabil Nurs.

[39] Takayama S (1997). A study of the process of change in cerebral disease patients’ recognition of their particular disabilities : a grounded theory approach. J Jpn Acad Nurs Sci.

[40] Da Silva CP, Carlegis M, Suchma K, Ostwald SK (2014). Falling, balance confidence, and fear of falling after chronic stroke. Phys Occup Ther Geriatr.

[41] Haines TP, Lee DCA, O’Connell B, Mcdermott F, Hoffmann T (2015). Why do hospitalized older adults take risks that may lead to falls?. Health Expect.

[42] Weerdesteyn V, De Niet M, Van Duijnhoven HJR, Geurts ACH (2008). Falls in individuals with stroke. J Rehabil Res Dev.

[43] Munford D, Gunn H (2020). What are the perceptions and experiences of falls amongst people with stroke who live in the community?. Disabil Rehabil.

[44] Schmid AA, Rittman M (2009). Consequences of poststroke falls: activity limitation, increased dependence, and the development of fear of falling. Am J Occup Ther.

[45] Pang MYC, Eng JJ (2008). Fall-related self-efficacy, not balance and mobility performance, is related to accidental falls in chronic stroke survivors with low bone mineral density. Osteoporos Int.

[46] Lutz BJ, Chumbler NR, Lyles T, Hoffman N, Kobb R (2009). Testing a home-telehealth programme for US veterans recovering from stroke and their family caregivers. Disabil Rehabil.

